# Associations between motor proficiency and academic performance in mathematics and reading in year 1 school children: a cross-sectional study

**DOI:** 10.1186/s12887-020-1967-8

**Published:** 2020-02-14

**Authors:** Kirstin Macdonald, Nikki Milne, Robin Orr, Rodney Pope

**Affiliations:** 1 Physiotherapy Program, Faculty of Health Sciences and Medicine, Bond University, Gold Coast, QLD 4229 Australia; 2 School of Community Health, Charles Sturt University, Thurgoona, NSW 2640 Australia

**Keywords:** Fine and gross motor skills, Physical activity, Academic performance, Mathematics, Reading

## Abstract

**Background:**

A key priority for learning during the early years of school is for children to develop skills in numeracy and literacy. Consequently, less time may be allocated in the curriculum to foster other important developmental areas, including the ongoing motor skill development of school children, which has been positively linked to academic performance. In order to promote holistic approaches to teaching and learning in the early years of school, it is necessary to further delineate the nature of associations between motor skills and foundation academic skills. The aim of this study was to examine associations between fine and gross motor proficiency and academic performance in mathematics and reading in Year 1 children.

**Methods:**

A cross-sectional study was conducted with Year 1 children from two primary schools in New South Wales, Australia (*N* = 55; 25 boys, 30 girls; mean age 6.77 ± 0.40 years). The Bruininks-Oseretsky Test of Motor Proficiency (2nd Edition) and the Wechsler Individual Achievement Test II (Australian Edition) were used to assess motor proficiency and academic performance in mathematics and reading, respectively. Associations between the components of motor proficiency and academic outcomes were examined using Pearson’s and Spearman’s correlation analyses. Hierarchical multiple linear regression analyses were conducted to determine how much variance in mathematics and reading composite scores could be explained by motor proficiency after controlling for age.

**Results:**

A significant moderate positive association was found between total motor composite and mathematics composite scores (*r* = .466, *p* < .001). Fine manual control composite scores were significantly associated with both mathematics (*r*_s_ = .572, *p* < .001) and reading (*r*_s_ = .476, *p* = .001) composite scores. After controlling for age, fine motor integration was the only component of motor proficiency that explained significant variance in mathematics and reading composite scores.

**Conclusions:**

The results of the study revealed that Year 1 children’s overall motor proficiency was significantly related to their mathematical ability. Children’s fine motor integration skills were also predictive of mathematics and reading ability. These study findings may interest both early childhood educators and paediatric health professionals.

## Background

The foundation for every child’s physical, cognitive, social and emotional development is laid during the early childhood period [1]. Success in these closely interrelated developmental domains during the early years is proposed to lead to positive health, education and social outcomes during adulthood [2]. In Australia, a key priority in the early years of school is for children to develop foundation skills in numeracy and literacy [3]. Consequently, there may be less time allocated in the curriculum to foster other important developmental areas, including the ongoing motor skill development of school children. Low competency in movement skills is reported to be associated with lower cardiorespiratory fitness and physical activity (PA) levels in Australian children and adolescents [4]. This is concerning, considering the physical health and socio-emotional benefits of children and young people’s participation in regular PA are well-established and important in the prevention of non-communicable diseases such as heart disease and stroke [5, 6].

Beyond the home environment, schools play an integral role in promoting the holistic development of students in the early years of school [3]. In fact, an expanding body of literature has identified that significant, positive relationships exist between PA, health and skill-related physical fitness, cognition and academic performance in children and adolescents, however, evidence for causality is yet to be determined [7, 8]. It has been proposed that coordination or perceptual-motor tasks and aerobic activities may differ in the way they affect the structure and function of the developing brain [9–11]. However, relationships between motor proficiency, cognition and academic performance have received less attention than relationships between the components of health-related physical fitness, such as cardiorespiratory fitness, cognition and academic performance [10].

Early studies investigating relationships between motor skill development and academic performance reported significant positive longitudinal associations between fine and gross motor composite scores assessed in Kindergarten and mathematics and reading achievement assessed in the later years of primary school [12–15]. A recently published systematic review by Macdonald et al. [16] found a strong level of evidence from observational studies to support significant positive associations between fine motor proficiency, particularly fine motor integration, and academic performance in mathematics and reading in children. There was also evidence, although weaker, to support several significant positive associations between academic performance and gross motor proficiency; specifically upper limb coordination, speed and agility and gross motor composite scores [16]. Associations between specific gross motor skills and academic outcomes have been investigated less extensively than associations between fine motor skills and academic outcomes in children in the early years of school, with the majority of studies reporting outcomes for gross motor composite scores or total motor composite scores (i.e. a combination of fine and gross motor skills) [13–15]. Consequently, inconsistent or insufficient findings have been reported regarding the relationships between several specific components of gross motor proficiency and academic performance in mathematics and reading [16].

Overall, a more comprehensive understanding of how the different components of gross motor proficiency are related to mathematics and reading skills in children in the early years of school is needed. Given this background, the aim of this study was to examine associations between fine and gross motor proficiency and academic performance in mathematics and reading in Year 1 school children. It was hypothesised that motor proficiency would be positively related to academic performance in mathematics and reading, however, it was anticipated that fine motor proficiency would be more strongly related to mathematics and reading outcomes than gross motor proficiency.

## Methods

### Setting and study design

This study was conducted in parallel with the Tweed Healthy Schools Project (THSP), an interprofessional clinical placement program for university health science students based in a school setting. A cross-sectional research design was employed, examining data collected at the start of the THSP. Ethics approval for the study was obtained from the Bond University Human Research Ethics Committee (Protocol number RO1836) and gatekeeper approval was granted by the State Education Research Approval Process in New South Wales, Australia (Reference number: 2014075). Parental consent was obtained in writing to confirm participation of each student involved in the study.

### Recruitment and study participants

Students from three mainstream Year 1 classes enrolled at two public primary schools in the northern region of New South Wales, Australia, were recruited from May to July, 2014 to participate in the study. Following gatekeeper approval from the principals at both schools, information sheets and consent forms were circulated to the parents of children across the three Year 1 classes. All students enrolled in the three Year 1 classes (*n* = 64) were invited and eligible to participate in the study provided their parents consented and the students themselves indicated assent. The study sample consisted of 55 Year 1 children (*n* = 25 boys; *n* = 30 girls; mean age = 6.77 ± 0.40 years, range = 5.42–7.75 years).

### Predictors, outcome measures and covariates

#### Motor proficiency

The Bruininks-Oseretsky Test of Motor Proficiency (BOT-2) Complete Form is a valid and reliable standardised motor assessment tool used for both clinical and research purposes [17]. The BOT-2 assesses the motor proficiency of individuals aged four to 21 years [17]. The tool measures fine and gross motor proficiency across eight individual subtests. Components of fine motor proficiency include fine motor precision (e.g. precise control of finger/hand movement), fine motor integration (e.g. precise control of finger/hand movement with the ability to integrate visual stimuli with motor control) and manual dexterity (e.g. reaching, grasping and bimanual coordination with small objects). The components of gross motor proficiency include upper limb coordination (e.g. visual tracking with coordinated arm and hand movement), bilateral coordination (e.g. body control, sequential and simultaneous coordination of the upper and lower limbs), balance (e.g. motor control skills integral for maintaining posture when standing, walking), running speed and agility (e.g. shuttle run, hopping and jumping over a balance beam) and strength (e.g. trunk, upper and lower body strength). Subtests may then be aggregated to yield four motor composites, including fine manual control (fine motor precision, fine motor integration), manual coordination (manual dexterity, upper limb coordination), body coordination (bilateral coordination, balance) and strength and agility (running speed and agility, strength). Due to differences in performance between girls and boys in subtests, sex and age-specific norms are used to interpret the scores of each assessment item. The total point score of each item was converted to scale scores for subtests and standard scores for motor composites. A total motor composite score was then calculated from the sum of standard scores for the motor composites. A standard score between 40 to 60 equates to a descriptive category of ‘average’ motor proficiency. Strong evidence for test-retest reliability (0.63–0.91 for ages 4–7 years), internal consistency (0.76–0.95 for mean age 4–7 years) and interrater reliability (0.86–0.99 for ages 4–21 years) has been reported for the BOT-2 [17, 18]. The BOT-2 is also deemed a valid test for evaluating motor proficiency, with scores able to differentiate between different clinical groups (e.g. groups with Developmental Coordination Disorder, Autism Spectrum Disorders) [17, 18].

#### Academic performance in mathematics and reading

The Wechsler Individual Achievement Test 2nd Edition (WIAT-II) Australian Standardised Edition is a valid and reliable test of academic performance [19]. The WIAT-II measures the achievement of individuals aged four to 85 years across the academic areas of reading, mathematics, written language and oral language [19]. The mathematics and reading composites were administered in this study, comprising five of the nine individual subtests in the achievement test. The mathematics composite included the maths reasoning and numerical operations (e.g. identifying and writing numbers) subtests. The reading composite included the word reading (e.g. phonological awareness and decoding skills), pseudoword decoding (e.g. phonetic decoding skills) and reading comprehension subtests. Standard scores were calculated based on participant age (in years and months) for each subtest, reading composite and mathematics composite. A standard score between 90 and 110 equates to a descriptive category of ‘average’ achievement. The age-based, inter-item reliability coefficients for the mathematics and reading subtests for children aged six and 7 years range between 0.79 and 0.98 [19]. The content, construct and criterion-related validity of the test have been investigated and correlations with other individually administered achievement tests are considered adequate [19]. The user level assigned to the WIAT-II restricts administration of the test to Allied Health (including physiotherapy) or Special Education professionals [20].

#### Covariates

Age, sex, ethnicity, school class and the Index of Community Socio-Educational Advantage (ICSEA) were measured as potential covariates. ICSEA is a scale of socio-educational advantage that takes into account the family background of school students, along with school level factors such as geographical location and student demographics [21]. The ICSEA is set at an average of 1000 with the lower the ICSEA value, the lower the level of educational advantage of students attending the school. Parents/caregivers were also asked to complete a questionnaire outlining any relevant medical history for their child, along with any reason why their child may not be able to participate in the study.

#### Procedure

Motor and academic assessments were conducted on separate days at the beginning of the third school term (July, 2014). All assessments took place during the regular school day, with permission from the classroom teacher. Prior to the commencement of the study, three physiotherapy and three exercise science university students were trained by a registered physiotherapist to administer the BOT-2. Under the supervision of a registered physiotherapist, physiotherapy and exercise science university students administered the BOT-2 Complete Form, which took approximately 40 to 60 min per participant. The WIAT-II test was individually administered by a registered physiotherapist who had completed the recommended training prior to administering the assessment tool and also had experience working with children. The test took approximately 45 to 60 min for each participant and took place in a quiet room to minimise the influence of distractions on performance.

### Statistical analysis

Statistical analyses for this study were conducted using the Statistical Package for the Social Sciences (Version 26) [22]. Descriptive statistics including mean, standard deviation (SD) and range were calculated for numerical variables including age, motor proficiency and academic performance. Frequencies (%) were calculated for categorical variables including sex and ethnicity. Normality of distributions and equality of variances were assessed to determine whether assumptions for parametric statistics were met. When variables did not meet the assumptions for using parametric tests, non-parametric statistical tests were employed. When assessing sex as a potential covariate for subsequent analyses examining relationships between motor proficiency and academic performance, independent samples t-tests were performed to determine any significant differences in mean age, academic scores and motor proficiency scores between girls and boys within the participant sample. Similarly, one way analyses of variance (ANOVA) with Bonferroni post hoc tests (using an overall alpha level of .05) were performed to determine any significant differences between the three classes and three ethnic groups in academic performance scores. Pearson’s and Spearman’s correlation analyses were performed to examine relationships between both age and academic performance scores. Pearson’s correlation analyses were subsequently used to examine the relationships between motor proficiency and academic performance in mathematics and reading for the total sample. Where assumptions of normality were not met, Spearman’s correlation analyses were performed on ranked data to analyse relationships between motor proficiency and academic performance variables. To account for multiple analyses of associations, Bonferroni corrections were applied to tests of significance in the correlational analyses. To describe the strength of correlation (r) between motor proficiency and academic performance variables, the rating guide described by Evans [23] was used as follows; *r* = 0.00–0.19 (very weak), *r* = 0.20–0.39 (weak), *r* = 0.40–0.59 (moderate), *r* = 0.60–0.79 (strong), and *r* = 0.80–1.0 (very strong). Finally, hierarchical multiple linear regression analyses were performed to determine how much variance in mathematics or reading composite scores (dependent variables) could be explained by each of the motor proficiency scale scores (independent variables) while controlling for covariates, enabling the relative predictive contribution of the components of motor proficiency to be assessed. Sensitivity analyses were conducted by repeating regression analyses with the removal of any outliers that were identified. To determine the effect size for the proportion of unique variance in academic performance explained by each predictor variable, Cohen’s f^2^ was calculated. According to Cohen’s [24] conventions, an effect size of .02 can be considered small, .15 can be considered medium and .35 can be considered large. A significance level of 5% (α = 0.05) was applied to all statistical tests, with Bonferroni corrections when appropriate. A statistical power analysis using G*Power 3 [25] indicated that the correlation analyses would have an 80% power to detect a correlation between two variables of 0.39 (a weak correlation) or greater if the sample numbered at least 50 participants, assuming an alpha level of 0.05.

## Results

**Fig. 1:**
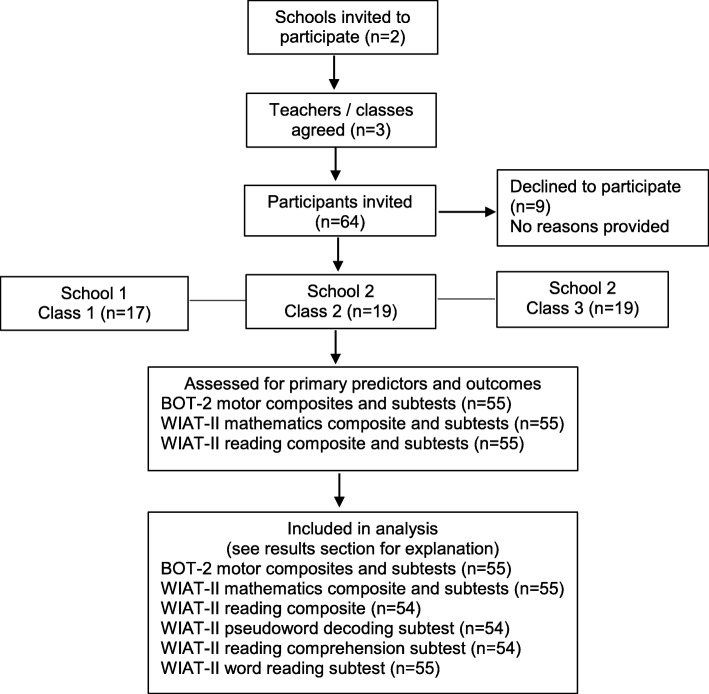
Flow of participants through the study

While 64 Year 1 children were invited to participate in the study, the parents of nine children did not provide consent for their child’s participation, leaving data for 55 children available for analysis. Figure 1 summarises the flow of participants through the study. Characteristics of the study participants are presented in Table 1.

**Table 1:** Characteristics of the Year 1 student participants

Characteristic		n (%)
Sex	Boys	25 (45.5)
	Girls	30 (54.5)
Ethnicity^a^	White	48 (87)
	Asian/Pacific Islander	3 (6)
	Other	4 (7)
ICSEA	ICSEA 965	38 (69)
	ICSEA 1011	17 (31)

*ICSEA* Index of Community Socio-Educational Advantage (average = 1000)

^a^Ethnicity classified according to the categories outlined in the BOT-2

Based on responses from parental/caregiver questionnaires, no child included in the present study had been previously diagnosed with an intellectual disability. Additionally, all children were drawn from and functioning in mainstream Year 1 classes.

### Motor proficiency and academic performance

The Australian normative scores for the pseudoword decoding subtest, reading comprehension subtest and the reading composite of the WIAT-II were only available for participants aged 5 years, 8 months and older, resulting in this data being unavailable for one participant (age = 5.42 years) and leaving data available for these subtests from 54 of the 55 participants (Fig. 1). Means, SD and ranges of performance data for the BOT-2 and WIAT-II (both by total sample and sex) are presented in Table 2.

**Table 2:** Mean, standard deviation (SD) and ranges for age, motor proficiency, mathematics ability and reading ability for all participants and separately for boys and girls

Measure	Total (*n* = 55)			Boys (*n* = 25)			Girls (*n* = 30)		
	Mean	SD	Range	Mean	SD	Range	Mean	SD	Range
Age (years)	6.77	0.40	5.42–7.75	6.81	0.31	6.25–7.42	6.74	0.46	5.42–7.75
Motor proficiency									
Fine motor precision^a^	13.67	3.83	3–24	13.88	3.79	5–24	13.50	3.92	3–22
Fine motor integration^a^	15.95	4.47	3–23	16.76	3.96	5–23	15.27	4.82	3–22
*Fine manual control*^*b*^	49.53	8.73	23–68	50.84	8.05	29–68	48.43	9.25	23–66
Manual dexterity^a^	14.25	4.22	4–21	15.32	3.90	5–21	13.37	4.33	4–19
Upper limb coordination^a^	16.31	6.13	1–28	16.56	5.80	4–25	16.10	6.49	1–28
*Manual coordination*^*b*^	50.98	10.12	24–66	52.40	9.59	30–64	49.80	10.55	24–66
Bilateral coordination^a^	17.58	4.01	5–24	18.76	3.63	8–22	16.60	4.11	5–24
Balance^a^	15.89	4.02	7–25	16.28	3.78	7–23	15.57	4.25	8–25
*Body coordination*^*b*^	54.49	9.11	32–70	56.64	8.49	34–70	52.70	9.36	32–68
Running speed and agility^a^	16.65	4.53	4–29	17.08	4.96	4–29	16.30	4.19	7–24
Strength^a^	13.67	4.92	4–26	13.60	4.64	5–26	13.73	5.22	4–24
*Strength and agility*^*b*^	49.87	9.49	29–80	50.72	10.21	29–80	49.17	8.95	29–65
*Total motor composite*^*b*^	51.56	10.65	22–79	53.40	10.54	27–79	50.03	10.67	22–70
Mathematics ability									
Maths reasoning^c^	93.84	16.33	51–144	97.80	17.06	51–144	90.53	15.20	63–116
Numerical operations^c^	94.67	14.96	61–140	95.56	15.93	66–140	93.93	14.33	61–131
*Mathematics composite*^*c*^	94.87	15.60	64–148	97.48	16.52	70–148	92.70	14.71	64–125
Reading ability									
Pseudoword decoding^c^ (*n* = 54)	96.61	15.00	71–127	97.88	15.99	75–126	95.52	14.29	71–127
Word reading^c^	100.27	16.84	69–132	100.28	17.69	70–131	100.27	16.40	69–132
Reading comprehension^c^ (*n* = 54)	97.09	13.52	62–127	96.92	15.31	62–127	97.24	12.04	66–124
*Reading composite*^*c*^ (*n* = 54)	97.96	16.70	66–132	98.88	18.35	67–132	97.17	15.43	66–127

^a^ Motor proficiency assessed by BOT-2: Normative sample scale score (M = 15, SD = 5, range = 1–35). Scores adjusted for chronological age (in years and months) and sex;

^b^ Motor proficiency assessed by BOT-2: Normative sample standard score (M = 50, SD = 10, range = 20–80). Scores adjusted for chronological age (in years and months) and sex;

^c^ Mathematics and reading ability assessed by WIAT-II Australian Edition: Normative sample standard score (M = 100, SD = 15, range = 40–160). Scores adjusted for chronological age (in years and months)

Overall the mean total motor composite standard score for the total sample was 51.56 ± 10.65 (range 22–79), which was considered ‘average’ motor proficiency. This was consistent with the mean of the normative sample [17], falling between ±1 SD for age and sex-specific norms (i.e. mean = 50, SD = 10, range 20–80). The mean mathematics composite standard score (94.87 ± 15.60, range 64–148) and mean reading composite standard score (97.96 ± 16.70, range 66–132) for the total sample were considered ‘average’ achievement. Each was slightly below but still within ±1 SD of the mean of the Australian normative sample (i.e. mean = 100, SD = 15, range 40–160) [19]. Finally, the mean total motor composite, mathematics composite and reading composite standard scores were categorised ‘average’ for both boys and girls (Table 2).

### Relationships between motor proficiency and mathematical skills

Overall, for the total sample, significant moderate positive correlations were found between mathematics composite scores and total motor composite (*r* = .466, *p* < .001) and fine manual control composite scores (*r*_s_ = .572, *p* < .001) (Table 3). Significant moderate positive correlations were evident between mathematics composite scores and the fine motor precision (*r*_s_ = .449, *p* = .001) and fine motor integration (*r*_s_ = .525, *p* < .001) subtests (Table 4). Significant moderate positive correlations were also found between the maths reasoning subtest and the fine motor precision (r_s_ = .449, *p* = .001), fine motor integration (*r*_s_ = .530, *p* < .001) and manual dexterity (*r*_s_ = .436, *p* = .001) subtests (Table 4). However, significant moderate positive correlations were only found for the numerical operations subtest with the fine motor integration subtest (*r*_s_ = .461, *p* < .001) (Table 4).

**Table 3:** Correlations between motor proficiency composites (standard scores) and mathematical and reading outcomes (standard scores) for total sample

	Fine manual control	Manual coordination	Body coordination	Strength and agility	Total motor composite
Mathematics composite (*n* = 55)	*r*_s_ = .572 (<.001)*	r = .399 (.003)	r_s_ = .296 (.028)	r = .389 (.003)	r = .466 (<.001)*
Maths reasoning (*n* = 55)	*r*_s_ = .587 (<.001)*	*r* = .382 (.004)	*r*_s_ = .264 (.051)	*r* = .354 (.008)	*r* = .448 (.001)*
Numerical operations (*n* = 55)	*r*_s_ = .472 (<.001)*	*r* = .382 (.004)	*r*_s_ = .264 (.051)	*r* = .387 (.004)	*r* = .439 (.001)*
Reading composite (*n* = 54)	*r*_s_ = .476 (.001)*	*r*_s_ = .299 (.028)	*r*_s_ = .142 (.307)	*r*_s_ = .180 (.193)	*r*_s_ = .316 (.020)
Pseudoword decoding (*n* = 54)	*r*_s_ = .438 (.001)*	*r*_s_ = .296 (.030)	*r*_s_ = .194 (.159)	*r*_s_ = .195 (.158)	*r*_s_ = .344 (.011)
Word reading (*n* = 55)	*r*_s_ = .521 (<.001)*	*r* = .319 (.017)	*r*_s_ = .154 (.263)	*r* = .215 (.115)	*r* = .362 (.007)
Reading comprehension (*n* = 54)	*r*_s_ = .395 (.003)	*r* = .284 (.037)	*r*_s_ = .029 (.832)	*r* = .144 (.298)	*r* = .270 (.048)

Spearman’s rho (r_s_) and Pearson’s (r) correlation coefficients are reported as appropriate

*p ≤ .05 (significant correlations after conducting Bonferroni correction at α = 0.05 / 35 = 0.0014)

**Table 4:** Correlations between motor proficiency subtests (scale scores) and mathematical and reading outcomes (standard scores) for total sample

	Fine motor precision	Fine motor integration	Manual dexterity	Upper limb coordination	Bilateral coordination	Balance	Running speed and agility	Strength
Mathematics composite (*n* = 55)	*r*_s_ = .449 (.001)*	*r*_s_ = .525 (<.001)*	*r*_s_ = .391 (.003)	*r* = .274 (.043)	*r*_s_ = .361 (.007)	*r* = .231 (.090)	*r* = .387 (.004)	*r* = .291 (.031)
Maths reasoning (*n* = 55)	*r*_s_ = .449 (.001)*	*r*_s_ = .530 (<.001)*	*r*_s_ = .436 (.001)*	*r* = .233 (.087)	*r*_s_ = .331 (.013)	*r* = .224 (.100)	*r* = .365 (.006)	*r* = .256 (.059)
Numerical operations (*n* = 55)	*r*_s_ = .357 (.007)	*r*_s_ = .461 (<.001)*	*r*_s_ = .306 (.023)	*r* = .296 (.028)	*r*_s_ = .331 (.014)	*r* = .217 (.111)	*r* = .380 (.004)	*r* = .293 (.030)
Reading composite (*n* = 54)	*r*_s_ = .368 (.006)	*r*_s_ = .470 (<.001)*	*r*_s_ = .254 (.064)	*r*_s_ = .289 (.034)	*r*_s_ = .259 (.058)	*r*_s_ = .163 (.240)	*r*_s_ = .252 (.065)	*r*_s_ = .162 (.241)
Pseudoword decoding (*n* = 54)	*r*_s_ = .311 (.022)	*r*_s_ = .459 (<.001)*	*r*_s_ = .250 (.069)	*r*_s_ = .279 (.041)	*r*_s_ = .353 (.009)	*r*_s_ = .156 (.261)	*r*_s_ = .251 (.068)	*r*_s_ = .188 (.174)
Word reading (*n* = 55)	*r*_s_ = .395 (.003)	*r*_s_ = .512 (<.001)*	*r*_s_ = .210 (.123)	*r* = .301 (.026)	*r*_s_ = .221 (.104)	*r* = .235 (.084)	*r* = .269 (.047)	*r* = .122 (.375)
Reading comprehension (*n* = 54)	*r*_s_ = .301 (.027)	*r*_s_ = .391 (.004)	*r*_s_ = .245 (.074)	*r* = .214 (.120)	*r*_s_ = .141 (.308)	*r* = .174 (.209)	*r* = .200 (.147)	*r* = .078 (.576)

Spearman’s rho (r_s_) and Pearson’s (r) correlation coefficients are reported as appropriate

*p ≤ .05 (significant correlations after conducting Bonferroni correction at α = 0.05 / 56 ≈ 0.001)

### Relationships between motor proficiency and reading skills

Significant moderate positive correlations were evident between the reading composite and fine manual control composite (*r*_s_ = .476, *p* = .001) (Table 3) and fine motor integration subtest (*r*_s_ = .470, *p* < .001) (Table 4). There were significant moderate positive correlations between the pseudoword decoding and word reading subtests and the fine manual control composite (*r*_s_ = .438, *p* = .001 and *r*_s_ = .521, *p* < .001 respectively) (Table 3) and fine motor integration subtest (*r*_s_ = .459, *p* < .001 and *r*_s_ = .512, *p* < .001 respectively) (Table 4).

### Covariates

Following consideration of a range of possible covariates, only participant age was included in subsequent regression analyses. Correlation analyses revealed a significant negative weak correlation between age (measured in years and months) and mathematics composite scores (*r* = −.327, *p* = .015) but a non-significant relationship between age and reading composite scores (*r*_s_ = −.197, *p* = .154). Although no such relationship was found for reading composite scores, given that age is known to be a key factor affecting scores on academic tests, it was included in subsequent regression analyses as a covariate wherever mathematics composite or reading composite scores were the dependent variables. No significant relationships between mathematics or reading composite scores and sex or school class or ICSEA or ethnicity were detected, and thus none of these were included as covariates in subsequent analyses.

### Predictors of academic performance in mathematics

To determine how much variance in mathematics performance could be explained by the components of motor proficiency beyond that accounted for by age, a hierarchical multiple regression analysis was conducted. Findings from the simple correlation analyses (Tables 3 and 4) were used to guide the motor proficiency variables that were entered into regression models, and these included fine motor integration and fine motor precision. Preliminary analyses found no violation of the assumptions of linearity, multicollinearity and homoscedasticity [26, 27]. However, a standardised residual greater than 3SD was found for one participant and was thus identified as an outlier and subsequently considered in a sensitivity analysis. Variables were entered into the model in the following steps: Age at step 1, fine motor precision at step 2 and fine motor integration at step 3 (Table 5). In combination, at step 3, the three predictor variables explained 34.7% of the variance in mathematics performance (*R*^2^ = .347, adjusted *R*^2^ = .309, ΔR^2^ = .094, F (3, 51) = 9.03, *p* < .001). By Cohen’s [24] convention, this was considered a large combined effect (f^2^ = 0.53). As can be seen in Table 5, in the final regression model, only fine motor integration (β = .430, *p* = .009) was a significant predictor of mathematics performance.

**Table 5:** Proportions of variance in (i) mathematical performance of Year 1 students that could be explained by fine motor precision and fine motor integration subtests, beyond that accounted for by age; (ii) reading performance of Year 1 students that could be explained by fine motor precision and fine motor integration subtests, beyond that accounted for by age

	R	R^2^	F, (df), p	Adj R^2^	ΔR^2^	ΔF, (df), p	B, [95% CI], SE B	β	t, p
Mathematics									
Step 1	.327	.107	6.37, (1, 53), **<.015**	.090	.107	6.37, (1, 53), **.015**	181.35, [112.49, 250.22], 34.34		5.28, **<.001**
Age							-12.77, [-22.92, -2.62], 5.06	-.327	-2.52, **.015**
Step 2	.503	.253	8.82, (2, 52), **.001**	.225	.146	10.18, (1, 52), **.002**	145.97, [78.58, 213.36], 33.58		4.35, **<.001**
Age							-10.72, [-20.19, -1.25], 4.72	-.275	-2.27, **.027**
Fine motor precision							1.57, [0.58, 2.56], 0.49	.386	3.19, **.002**
Step 3	.589	.347	9.03, (3, 51), **<.001**	.309	.094	7.31, (1, 51), **.009**	124.19, [58.50, 189.88], 32.72		3.80, **<.001**
Age							-8.65, [-17.72, 0.43], 4.52	-.222	-1.91, .061
Fine motor precision							0.39, [-0.89, 1.67], 0.64	.096	0.61, .544
Fine motor integration							1.50, [0.39, 2.61], 0.55	.430	2.70, **.009**
Reading									
Step 1	.202	.041	2.21, (1, 52), .143	.022	.041	2.21, (1, 52), .143	162.04, [75.40, 248.68], 43.18		3.75, **<.001**
Age							-9.43, [-22.16, 3.30], 6.34	-.202	-1.49, .143
Step 2	.387	.150	4.50, (2, 51), .**016**	.117	.109	6.56, (1, 51), **.013**	124.47, [36.98, 211.97], 43.58		2.86, **.006**
Age							-6.82, [-19.10, 5.46], 6.12	-.146	-1.12, .270
Fine motor precision							1.45, [0.31, 2.59], 0.57	.335	2.56, **.013**
Step 3	.502	.252	5.60, (3, 50), **.002**	.207	.102	6.79, (1, 50), **.012**	109.41, [25.65, 193.18], 41.71		2.62, **.012**
Age							-5.81, [-17.47, 5.86], 5.81	-.124	-1.00, .322
Fine motor precision							0.10, [-1.40, 1.60], 0.75	.022	0.13, .898
Fine motor integration							1.68, [0.39, 2.98], 0.65	.450	2.61, **.012**

Unstandardised (B) and standardised (β) regression coefficients; significant *p*-values (*p* < .05) in bold

A sensitivity analysis was performed to examine whether results from this hierarchical multiple regression analysis were influenced by the outlier in the sample (Table 6). Following removal of the outlier, in combination, at step 3, the three predictor variables explained 39.2% of the variance in mathematics performance (*R*^2^ = .392, adjusted *R*^2^ = .355, ΔR^2^ = .084, F (3, 50) = 10.73, *p* < .001). By Cohen’s [24] convention, this was considered a large combined effect (f^2^ = 0.64). When the outlier was removed from the sample, fine motor integration (β = .407, *p* = .012) and age (β = −.244, *p* = .036) were both significant predictors of mathematics performance (Table 6).

**Table 6:** Proportions of variance in mathematical performance of Year 1 students that could be explained by fine motor precision and fine motor integration subtests, beyond that accounted for by age following removal of outlier

	R	R^2^	F, (df), p	Adj R^2^	ΔR^2^	ΔF, (df), p	B, [95% CI], SE B	β	t, p
Mathematics									
Step 1	.352	.124	7.37, (1, 52), .**009**	.107	.124	7.37, (1, 52), **.009**	176.19, [115.24, 237.14], 30.37		5.80, <**.001**
Age							-12.15, [-21.13, -3.17], 4.48	-.352	-2.71, **.009**
Step 2	.555	.308	11.35, (2, 51), **<.001**	.281	.184	13.56, (1, 51), **.001**	141.13, [83.17, 199.10], 28.87		4.89, <**.001**
Age							-10.12, [-18.26, -1.98], 4.06	-.293	-2.50, **.016**
Fine motor precision							1.56, [0.71, 2.41], 0.42	.433	3.68, .**001**
Step 3	.626	.392	10.73, (3, 50), **<.001**	.355	.084	6.87, (1, 50), **.012**	123.13, [66.51, 179.76], 28.19		4.37, <**.001**
Age							-8.42, [-16.24, -0.59], 3.90	-.244	-2.16, **.036**
Fine motor precision							0.57, [-0.54, 1.67], 0.55	.157	1.03, .310
Fine motor integration							1.26, [0.30, 2.23], 0.48	.407	2.62, .**012**

Unstandardised (B) and standardised (β) regression coefficients; significant *p*-values (*p* < .05) in bold

### Predictors of academic performance in reading

To determine how much variance in reading performance could be explained by the components of motor proficiency beyond that accounted for by age, a separate hierarchical multiple regression analysis was conducted. The two motor proficiency subtests most significantly correlated with reading composite scores were fine motor precision and fine motor integration (Table 4). Variables were entered into the model in the following steps: Age at step 1, fine motor precision at step 2 and fine motor integration at step 3 (Table 5). In combination, at step 3, the three predictor variables explained 25.2% of the variance in reading performance (*R*^2^ = .252, adjusted *R*^2^ = .207, ΔR^2^ = .102, F (3, 50) = 5.60, *p* = .002). By Cohen’s [24] convention, this was considered a medium combined effect (f^2^ = 0.34). As can be seen in Table 5, in the final regression model, fine motor integration (β = .450, *p* = .012) was the only significant predictor of reading performance.

## Discussion

The purpose of this study was to examine associations between fine and gross motor proficiency and academic performance in mathematics and reading in Year 1 school children. Several key findings were evident. Firstly, significant moderate positive correlations were found between total motor composite and mathematics composite scores. Secondly, the fine manual control composite was significantly associated with both mathematics and reading composite scores. Finally, after controlling for age, fine motor integration was the only component of motor proficiency that was a significant predictor of mathematics and reading composite scores. The combined effect also appeared to be larger for mathematics (f^2^ = 0.53) than reading (f^2^ = 0.34) suggesting that fine motor integration skills may have a stronger association with mathematics than reading performance. Collectively, the findings from this study highlight the importance of educators promoting the holistic development of students, including their motor skill development, in early primary school classrooms.

### Fine motor proficiency, mathematics and reading ability

Findings from the present study are consistent with other cross-sectional research examining associations between fine motor proficiency, mathematics and reading skills in Year 1 children [28, 29]. For example, Pienaar et al. [28] found that visual motor integration skills were more strongly associated with mathematics and reading performance than total motor proficiency in a large sample of socio-economically disadvantaged first grade learners from South Africa. Significant medium to strong correlations between the maths reasoning subtest of the WIAT-II and the fine motor precision (*r* = .597, *p* < .001) and fine motor integration (*r* = .569, *p* < .001) subtests of the BOT-2 have also been reported in a small sample of Year 1 children in the UK [29]. However, similar to the findings in the present study, significant correlations were only found between the word reading and fine motor integration subtests (*r* = .377, *p* = .003), but not fine motor precision (*r* = .198, *p* = .129) in the sample of Year 1 children in the UK [29].

Analyses conducted in the present study revealed that after accounting for age and fine motor integration, fine motor precision was not a significant predictor of mathematics and reading composite scores in this sample of Year 1 children. This is consistent with other studies that have specifically evaluated relationships between academic performance in mathematics and reading and the individual components of fine motor skills, including fine motor integration (or visual motor integration), fine motor precision and manual dexterity (or fine motor manipulation / coordination) [30–32]. For example, a longitudinal study by Kim et al. [32] found that fine motor coordination and visual motor integration were related to the mathematical ability of students in Kindergarten, however, only visual motor integration was related to mathematical skills in the same sample of students when they reached Year 1. The authors suggested that children’s mastery over fine motor coordination skills may explain why they were no longer related to children’s mathematical skills in Year 1 [32].

It was beyond the scope of this study to ascertain the underlying mechanisms that may explain the observed study findings. However, one potential explanation as to why fine motor integration may be more strongly related to mathematics and reading in Year 1 children than other fine motor skills (i.e. fine motor precision and manual dexterity) has been proposed in the literature, and relates to the notion of automaticity [32, 33]. Motor and cognitive processes (such as executive functions) may share similar neural pathways in the brain with researchers conducting functional neuroimaging studies demonstrating that when tasks are novel or complex, the cerebellum and pre-frontal cortex are both activated [34]. Motor tasks appear to become more automatic with practice leading to a reduction of activity in these two regions [34, 35].

### Gross motor proficiency, mathematics and reading ability

Previous studies examining relationships between gross motor composite scores and mathematical skills in children in the early years of school (e.g. pre-kindergarten to Year 2) have reported significant very weak to moderate positive associations [16]. However, few studies have previously investigated relationships between the individual components of gross motor proficiency and academic performance in mathematics in Year 1 children, like the present study [16, 36]. Overall, the components of gross motor proficiency that were most strongly related to mathematics composite scores were running speed and agility and bilateral coordination, though these relationships did not reach statistical significance after adjusting for multiple comparisons. The lack of significant findings are thus in contrast to those reported in the systematic review by Macdonald et al. [16] who found a strong level of evidence to support significant very weak to weak positive associations between speed and agility and mathematical ability in studies conducted with slightly older children aged nine to 13 years.

Significant very weak to moderate positive correlations between gross motor composite scores and reading skills in children in the early years of school have also previously been reported [16]. In the present study, upper limb coordination appeared to be the component of gross motor proficiency most strongly related with reading composite scores, particularly pre-reading skills including word reading and pseudoword decoding, but these relationships did not reach statistical significance. Again, the lack of significant findings are in contrast with the systematic review by Macdonald et al. [16] who found evidence to support significant weak positive associations between upper limb coordination and reading ability, including in Kindergarten children [37], students in Year 5 [38] and adolescents [39, 40].

### Limitations

Several limitations are important to acknowledge in this study. Firstly, due to the cross-sectional design, the results cannot infer causality nor provide evidence of the underlying mechanisms for observed associations between motor proficiency and academic performance in mathematics and reading in this cohort of Year 1 children. Secondly, a relatively small sample size (*n* = 55) was included in the study, however, this was pre-determined by the study being conducted in parallel with the THSP. This may have limited the statistical power of the study to detect relationships between variables reflecting smaller effect sizes. Thirdly, variables including cognitive skills (e.g. IQ, executive functions such as working memory, inhibitory control, cognitive flexibility), measures of health-related fitness (e.g. body mass index, cardiorespiratory fitness) and PA levels were not assessed and thus not taken into account. Finally, the cohort of Year 1 children came from two public primary schools in the same region of Australia and thus caution should be applied in generalising the findings to other regions or schools with a different school ICSEA status.

## Conclusion

The collective findings from this study revealed several significant positive relationships between motor proficiency and academic performance, particularly in mathematics, in this cohort of Year 1 children. Specifically, Year 1 children’s overall motor proficiency was significantly related to their mathematical skills. Additionally, children’s fine motor integration skills were predictive of their mathematical and reading ability. The results of this study may interest both early childhood educators and paediatric health professionals. For example, knowledge of associations between motor skills and academic outcomes may prompt educators to identify early, for further investigation, any children with poorly developed or delayed motor skills as they transition to school. Finally, study findings may be useful in guiding the future design of fine and gross motor skill interventions for children in the early years of school to evaluate more rigorously their impact on foundation scholastic skills.

## Data Availability

The dataset analysed during the current study may be available from the corresponding author on reasonable request and following approval from the Ethics Committee.

## Abbreviations

BOT-2: Bruininks Oseretsky Test of Motor Proficiency
ICSEA: Index of Community Socio-Educational Advantage
PA: Physical activity
THSP: Tweed Healthy Schools Project
WIAT-II: Wechsler Individual Achievement Test
